# Pressure-induced magnetic moment abnormal increase in Mn_2_FeAl and non-continuing decrease in Fe_2_MnAl via first principles

**DOI:** 10.1038/s41598-017-16735-1

**Published:** 2017-11-28

**Authors:** Yang Ze-Jin, Gao Qing-He, Xiong Heng-Na, Shao Ju-Xiang, Wang Xian-Wei, Xu Zhi-Jun

**Affiliations:** 1 School of Science, Zhejiang University of Technology, Hangzhou, 310023 China; 2 Ames Laboratory, Department of Energy and Department of Physics and Astronomy, Iowa State University, Ames, Iowa 50011 United States; 3 College of Science, Northeastern University, Shenyang, 110004 China; 4 Information Engineering College, Liaoning University of Traditional Chinese Medicine, Shenyang, 110847 China; 5 Computational Physics Key Laboratory of Sichuan Province, Yibin University, Yibin, 644000 China

## Abstract

The magnetism of Fe_2_MnAl and Mn_2_FeAl compounds are studied by first principles. Evolutions of magnetic moment of Fe_2_MnAl display distinct variation trends under pressure, showing three different slopes at different pressure intervals, 0~100 GPa, 100~250 GPa, 250–400 GPa, respectively, and the moment collapses finally at 450 GPa. The magnetic moment of Mn_2_FeAl shows an increasing tendency below 40 GPa and decreases subsequently with pressure, and collapses ultimately at about 175 GPa. Such non-continuing decrease of Fe_2_MnAl originates from the unusual charge transfer of Fe and Mn and bond populations rearrangement of Fe-Fe and Mn-Fe, whereas the distinct moment evolution of Mn_2_FeAl is attributed to the complicated distributions of bond populations. The half-metallicity of the compounds can be maintained at low pressure, below about 100 GPa in Fe_2_MnAl and 50 GPa in Mn_2_FeAl. The magnetic moment collapse process didn’t induce volume and bond length anomalies in the two compounds, the unique anomaly is the elastic softening behaviour in elastic constant *c*_44_ and shear (*G*) and Young’s (*E*) moduli of Fe_2_MnAl at 270 GPa, where the second moment collapse occurs.

## Introduction

The presence of nanotechnology requires more novel materials with extraordinary physical properties. Half-metallic heusler magnetic compounds can play a key role in the field of microdevice as it shows metallic properties in one of its spin orientations while an evident energy gap is formed in the other spin orientation. The number of heusler family is more than 1000 members and nearly all of them crystalline similar to that of binary semiconductors^1^. The general chemical formula can be classified into two different styles, half- (semi-) or full-heusler structures.

Recently, half-metallic (XYZ) characteristic has been reported in full-Heusler (X_2_YZ) alloys, including Co_2_YZ, Mn_2_YZ, Fe_2_YZ, Cr_2_YZ, and V_2_YZ^2–5^, where X and Y are transition metal elements and Z is a *sp* element, in which Fe-(Mn-)containing compounds attract much attention due to the complicated magnetic behavior of Fe and Mn element. Fe_2_MnAl exhibits Cu_2_MnAl-type structure ($Fm\bar{3}m$, 225#) and Mn_2_FeAl has the Hg_2_CuTi-type structure ($F\bar{4}3m$, 216#). However, the detailed magnetic moment evolution under pressure for the typical Fe-(Mn-)containing compounds are still unknown, in particular for the key role of the on-site coulomb term in this kind of compounds.

In this paper, two representative compounds Fe_2_MnAl and Mn_2_FeAl are deeply studied under pressure by first principles. Our comprehensive calculations confirmed the crucial role of on-site coulomb term in the investigation of electronic structures, whereas such influence is not sensitive in the macroproperty calculations. In addition, we also systematically simulated the magnetic moment evolution with volume variations and found the distinctly non-continuing decrease of moment in Fe_2_MnAl. Meanwhile, the abnormally magnetic moment increase with pressure is also observed in Mn_2_FeAl.

## Computational Methods

Spin-polarized geometric and electronic relaxations are performed by the projector augmented wave method^6^. The exchange correlation is calculated using generalized gradient approximation perdew-burke-ernzerh function (GGA-PBE)^7^. The k meshes^8^ 9 × 9 × 9 is used for the first Brillouin zone integration. Energy cutoff 450 eV is set for plane wave basis. The on-site Coulomb term *U* is selected for Fe (*U* = 2.0 eV) and Mn (*U* = 0.8 eV). The exchange integral *J* = 1.1 eV is also carefully selected. The self-consistent convergence of the energy is at 5.0 × 10^−7^ eV/atom.

## Results and Discussion

### Structural properties

**Figure 1:**
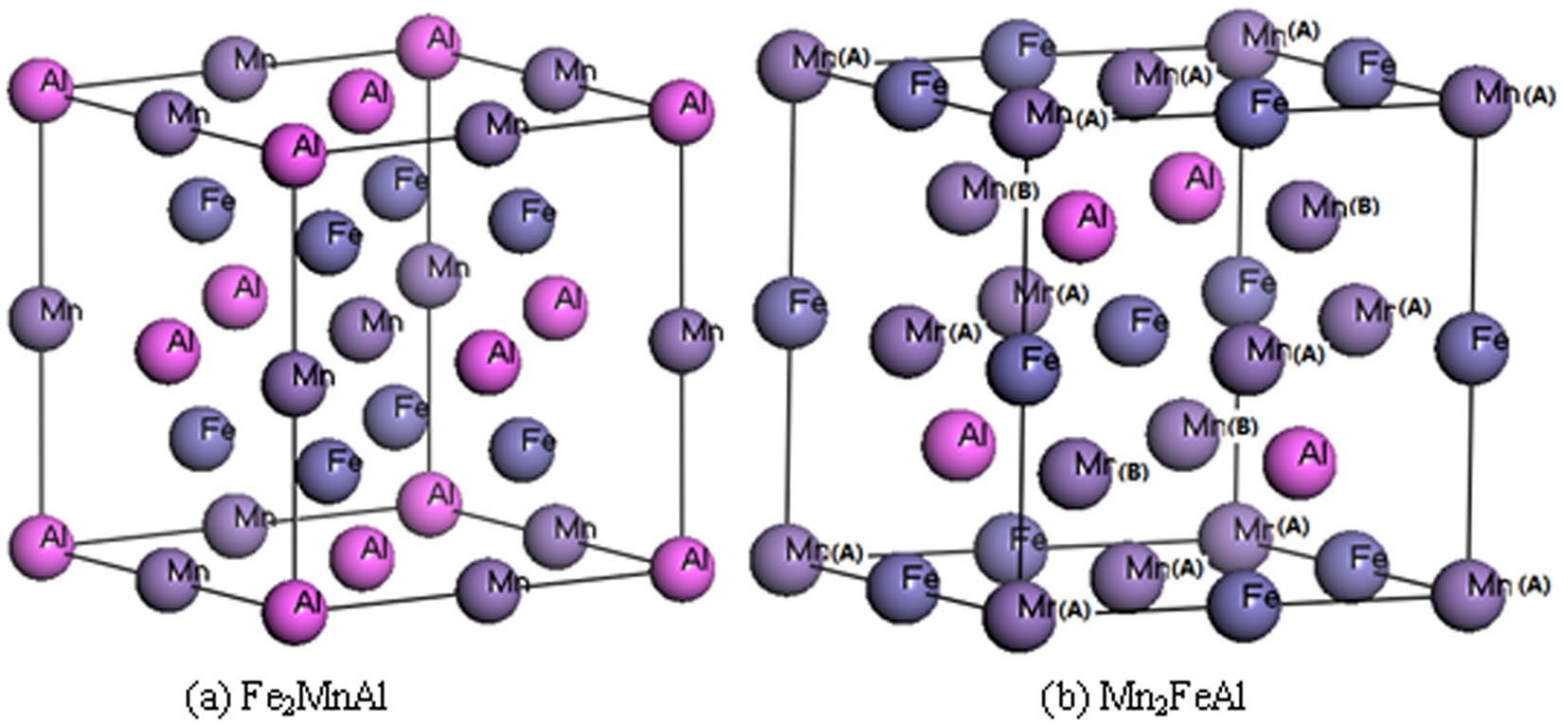
(**a**) The atomic coordinates are (0, 0, 0), (0.5, 0.5, 0.5), and (0.25, 0.25, 0.25) for Al, Mn, and Fe, respectively in Fe2MnAl. (**b**) Mn atoms site at A (0, 0, 0,) and B (0.25, 0.25, 0.25) denoted by Mn(**A**) and Mn(**B**), Fe and Al atoms site at (0.5, 0.5, 0.5) and Al (0.75, 0.75, 0.75) in Mn2FeAl, respectively.

The coordinates of the two compounds are shown in Fig. 1. The calculated volume-energy relationship determines the ferromagnetic (FM) states as the ground states of the Fe_2_MnAl and Mn_2_FeAl. The calculated equilibrium lattice constants are 5.673 and 5.781 Å for Fe_2_MnAl and Mn_2_FeAl, respectively, in good agreement with previous results 5.64^9^, 5.683^10^, 5.67^11^, 5.85^12^, 5.816^13^ and 5.76^10^, 5.725^14^, 5.74^15^, respectively. All of the bonds in Mn_2_FeAl are longer than their counterparts in Fe_2_MnAl, such as the lengths of Mn(A)-Fe and Mn(B)-Fe are longer than those of Fe-Fe and Mn-Fe, respectively. There are two different kinds of bonds in each compound, the respective lengths are 2.8368 and 2.4568 Å in Fe_2_MnAl, slightly shorter than those of 2.8904 and 2.5032 Å in Mn_2_FeAl, respectively.

### Elastic constants

**Figure 2:**
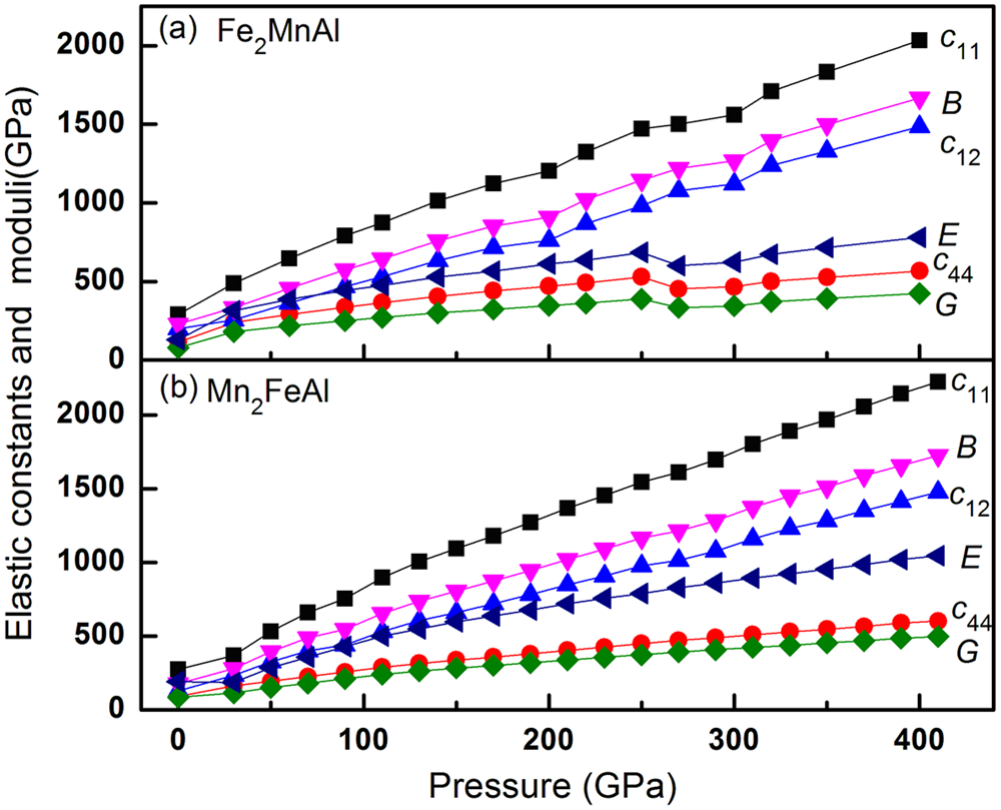
The elastic constants and mechanical moduli of Fe_2_MnAl and Mn_2_FeAl.

**Figure 3:**
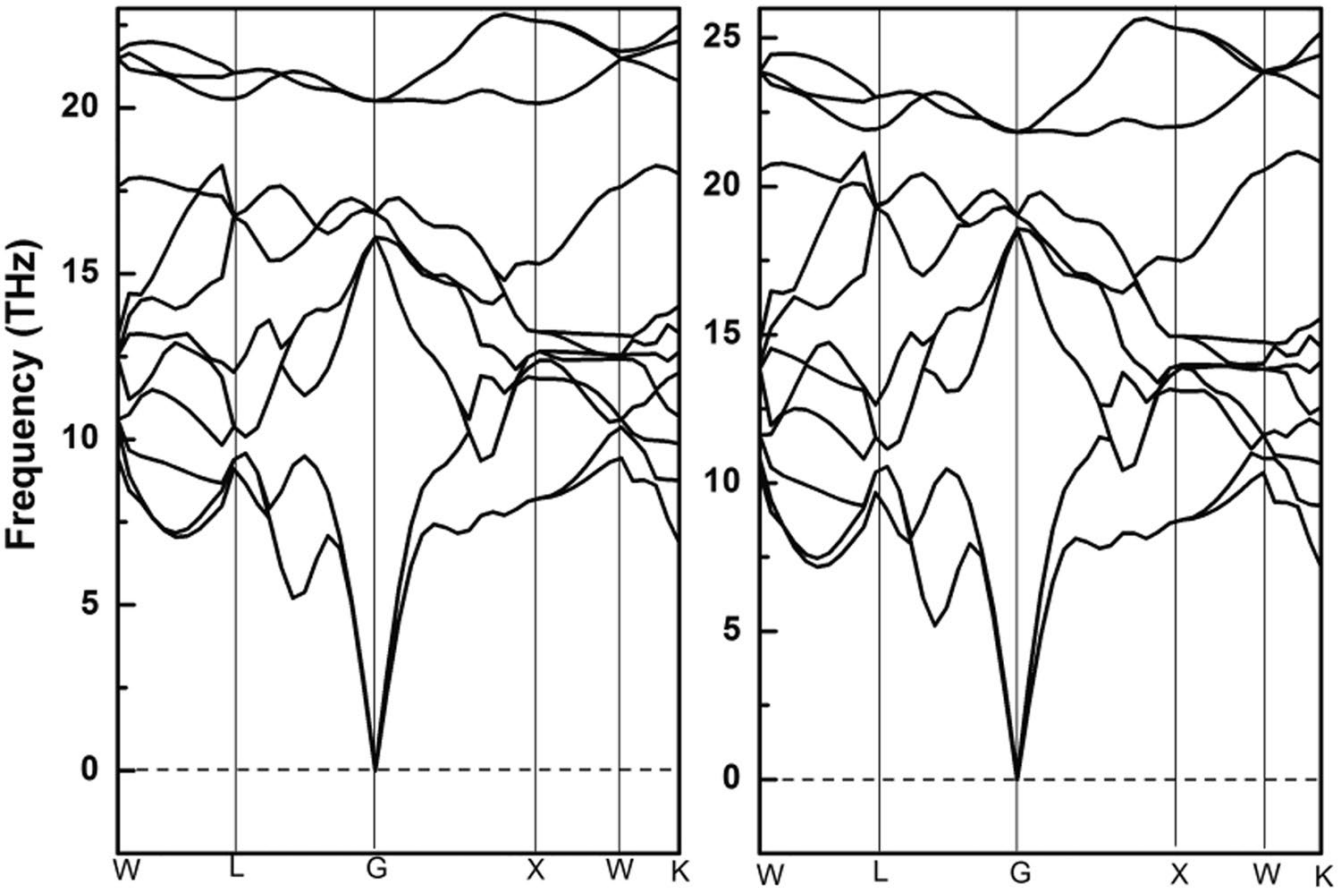
Phonon dispersion curves of Fe_2_MnAl at 270 and 400 GPa, respectively.

We obtain the three independent elastic constants, namely *c*_11_, *c*_12_ and *c*_44_, with respective values of 290.3, 197.9, 113.9 GPa for Fe_2_MnAl, and 272.3, 126.7, 91.6 GPa for Mn_2_FeAl. Clearly, the respective values of Fe_2_MnAl are larger than those of Mn_2_FeAl. The calculated Young’s (*E*) and shear (*G*) moduli of Fe_2_MnAl (129.9, 79.3 GPa) are substantially smaller than those of Mn_2_FeAl (191.7, 83.5 GPa), whereas this is not true in bulk modulus (*B*), with values of 228.7 GPa in Fe_2_MnAl and 175.2 GPa in Mn_2_FeAl, both agreeing well with respective literatures 215.9^9^, 200.9^10^, 210.1^16^ GPa and 150.2^10^ GPa, respectively. The anisotropy factor *A* = 2*c*_44_/(*c*_11_-*c*_12_) is 2.46 for Fe_2_MnAl, agreeing well with the available data 2.34^4^, suggesting the present elastic constants are reliable. The calculated elastic constants satisfy the stability criteria^17^(*c*_11_-*c*_12_ > 0, *c*_11_ > 0, *c*_44_ > 0, *c*_11_ + 2*c*_12_ > 0, *c*_12_ < B* < c*_11_) in a wide pressure range, as shown in Fig. 2. To test this result, we simulate the phonon dispersion curves at 270 and 400 GPa by the finite displacement methodology with a supercell volume of eight times larger than the unit cell (128 atoms in total), as shown in Fig. 3. Clearly, the softening behaviors of shear (*c*_33_, *G*) and Young’s (*E*) moduli of Fe_2_MnAl at 270 GPa failed to induce the structural instability at such critical point.

The magnitude of *B/G* ratio^18^ greater than 1.75 meaning the crystal is brittle, otherwise it means ductile behavior. Our computed *B/G* ratios are 2.882 and 2.097 for Fe_2_MnAl and Mn_2_FeAl, respectively. The simulated Poisson’s ratios (*σ*), 0.345 for Fe_2_MnAl and 0.294 for Mn_2_FeAl, indicate that Fe_2_MnAl displays stronger metallic bonding character. The relationship between bulk (*B*) and shear (*G*) moduli are *G* = 1.1*B* and *G* = 0.6*B* for covalent and ionic materials, respectively. Our simulated *G*/*B* ratios are 0.347 and 0.477 for Fe_2_MnAl and Mn_2_FeAl, respectively, indicating the weak ionic bonding feature.

### Electronic properties

**Figure 4:**
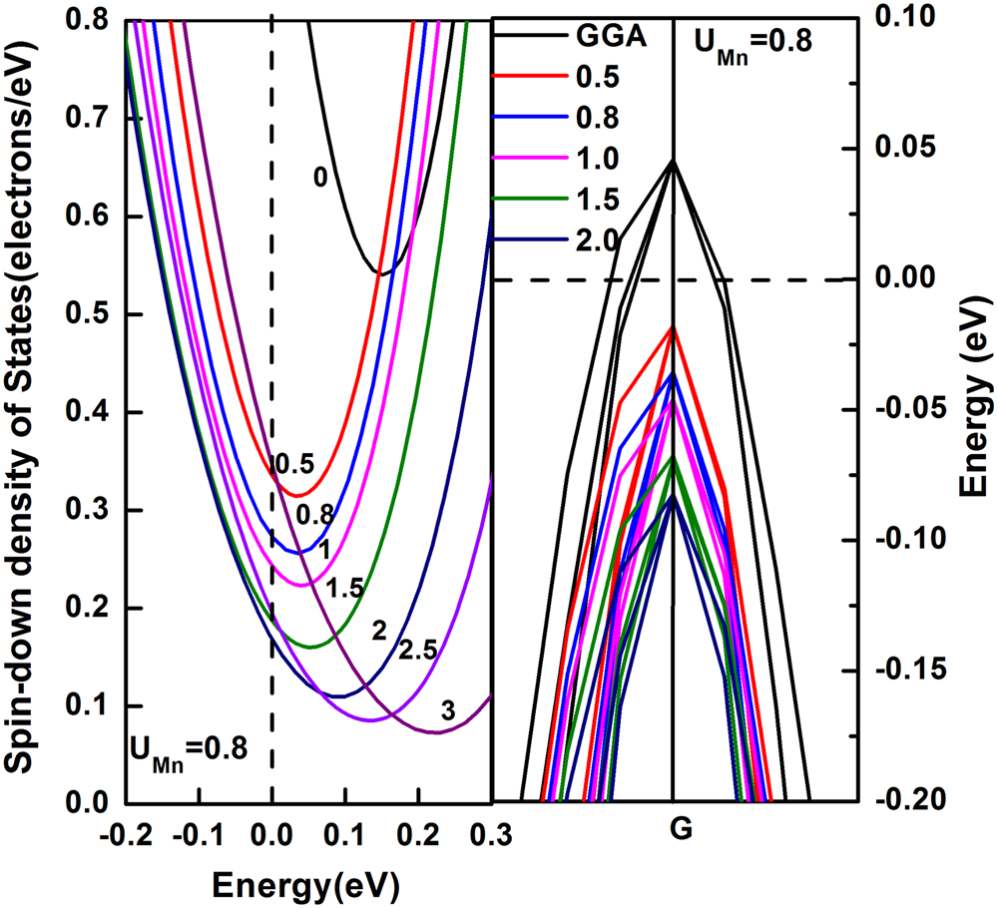
Determination of the used on-site Coulomb parameter *U*, the optimal values are 0.8 eV and 2.0 eV for Mn and Fe, respectively.

Our thorough test found that it is necessary to introduce the on-site coulomb term *U* and *J* during the calculation of electronic properties, otherwise the band structures at spin-down orientation will cross the Fermi level, as is shown in Fig. 4, in which the left panel is the density of states (DOS) but the right one is the energy band at G point of the Brillouin zone of Fe_2_MnAl near Fermi level in the spin-down orientation. According to the definition of spin polarization given^19^ by *P* = [*N*_↑_(*E*_*F*_) − *N*_↓_(*E*_*F*_)]/*N*_↑_(*E*_*F*_) + *N*_↓_(*E*_*F*_)], where *N*_↑_(*E*_*F*_) and *N*_↓_(*E*_*F*_) correspond to the spin-up and spin-down DOS at Fermi level, respectively. Considering the largest spin polarization nature^10^ and the largest energy gap between conduction band bottom and valence band top, and the best agreement of the calculated magnetic moment with the available calculations, the optimal combinations should be *J* = 1.1 eV and *U* = 2.0 and 0.8 eV for Fe and Mn, respectively. The calculated gaps are 0.55 and 0.4 eV for Fe_2_MnAl and Mn_2_FeAl, respectively, differing with the results^10^ 0.465 (Fe_2_MnAl) and 0.544 eV (Mn_2_FeAl) by ignoring the influence of the on-site coulomb term. Neglect of the influence of on-site coulomb term^11^ couldn’t obtain the half-metallic character for Fe_2_MnAl and Fe_2_MnP.

**Figure 5:**
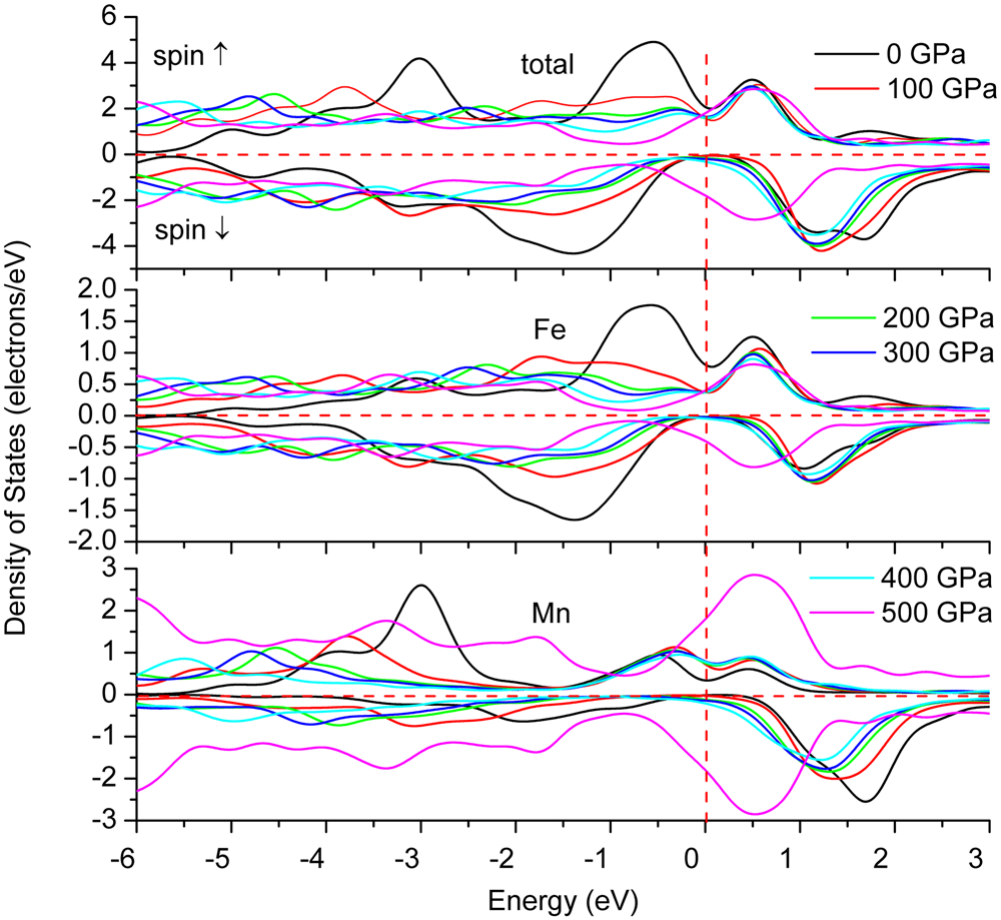
Spin-dependent total (one unit cell) and atomic density of states of Fe_2_MnAl.

**Figure 6:**
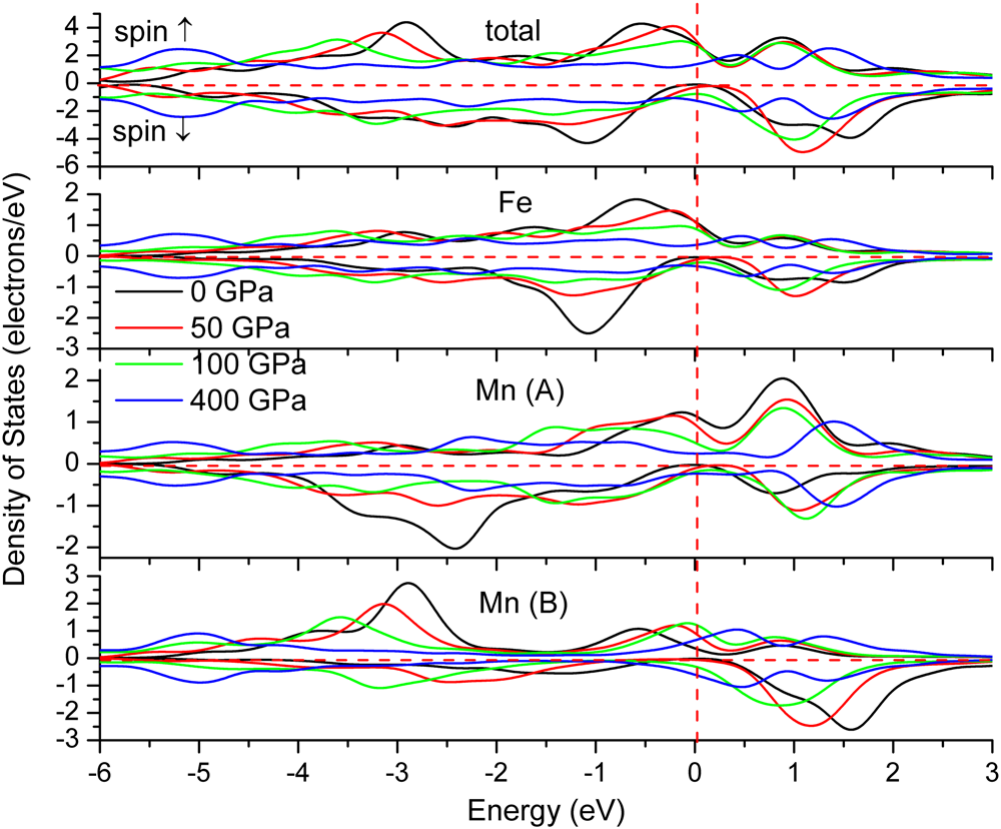
Spin-dependent total (one unit cell) and atomic density of states of Mn_2_FeAl.

To further illustrate the nature of electronic structures, we simulate the total and atomic DOS, as are shown in Figs 5 and 6. Deep analysis to the DOS for the two compounds found that *d* states of Fe and Mn distributed mainly at the both sides of Fermi level, with an energy range of −4.5~5 eV, in which Mn displays stronger magnetism than Fe owing to its less overlap between spin-up and spin-down channels. The DOS profile also clearly reflects the strong magnetism of Mn and weak magnetism of Fe. Furthermore, both the two compounds formed potential valleys at Fermi level, implying the extreme stability of the compounds, as is reflected in the moment collapse process.

**Figure 7:**
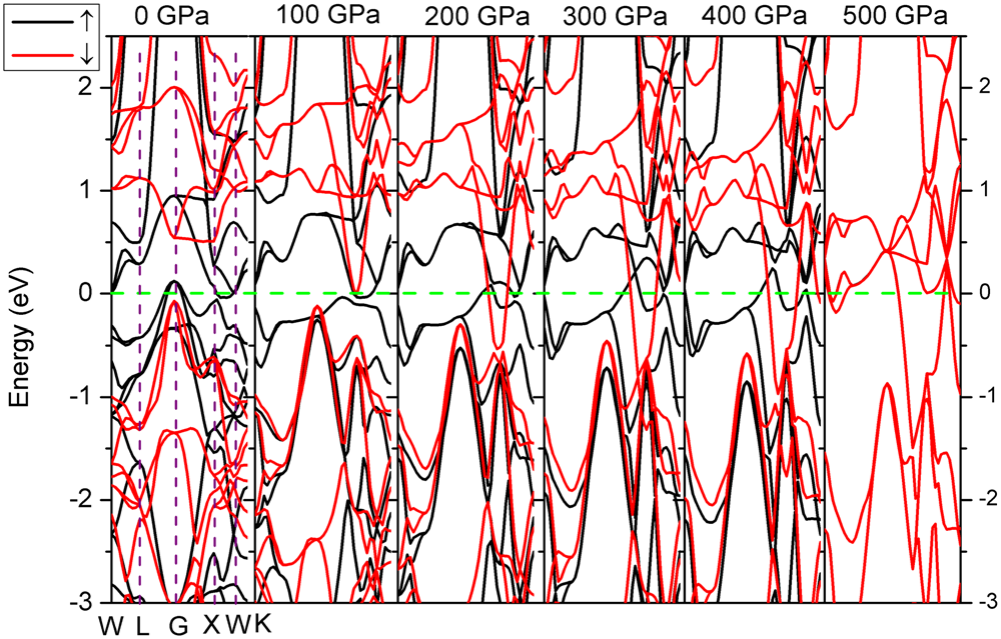
Spin-polarized band structures of Fe_2_MnAl. The Fermi level is set to zero (dash horizontal green line). Only the high symmetry points in the first Brillouin zone at 0 GPa are shown for simplicity.

**Figure 8:**
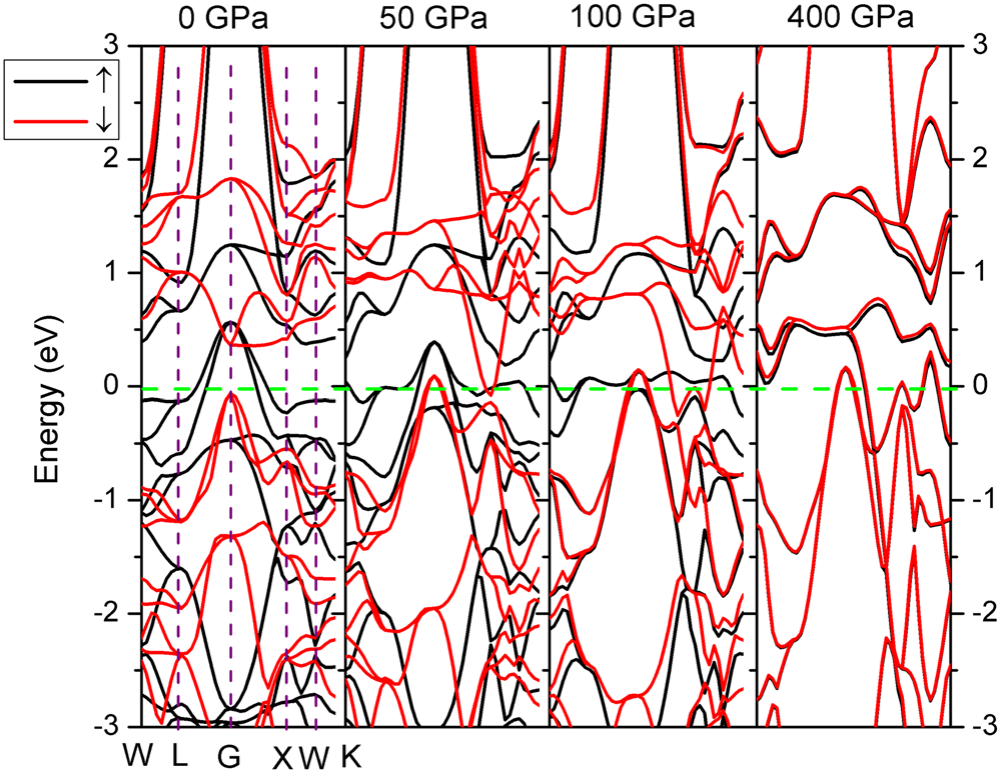
Spin-polarized band structures of Mn_2_FeAl, same definition is used with that of Fig. 7.

The two different kinds of Mn atoms in Mn_2_FeAl, named Mn(A) and Mn(B), presenting almost opposite charge probability distributions in the whole space, namely, charges located at spin-up channel distribute mainly at Fermi level, whereas charges located at spin-down channel distribute mainly at low-energy zone in Mn(A), which is just opposite in Mn(B) atom. The substantial discrepancy of spin-up and spin-down charge location is the origin of the higher magnetism collapse pressure. Mn(B) bonds with more Al and thus accumulates more charges at lower energy zone, which is obviously larger than that of Mn(A) in Mn_2_FeAl, as are also the cases for their Fermi-level values. In particular, Mn(A) exhibits a peak at Fermi level but Mn(B) and Fe locate at the steep hill, both of which make the charge transfer easier with respect to that of Fe_2_MnAl, consisting with the fact of its lower magnetism collapse pressure. Cr ion moment collapse process has been deeply discussed in Cr_2_TiAlC_2_^20^, in which the contrary charge shift direction causes the moment collapse, whereas the present moment collapse of Fe and Mn show only certain shift under pressure. The DOS profiles of Fe_2_MnAl and Mn_2_FeAl consists well with their energy band profiles, as shown in Figs 7 and 8. The two bands of spin-up and spin-down channels of Mn_2_FeAl are extremely difficult to be totally merged even at 400 GPa due mainly to the subtle difference of conduction band profiles of Mn(A) and Mn(B).

### Magnetic properties

**Figure 9:**
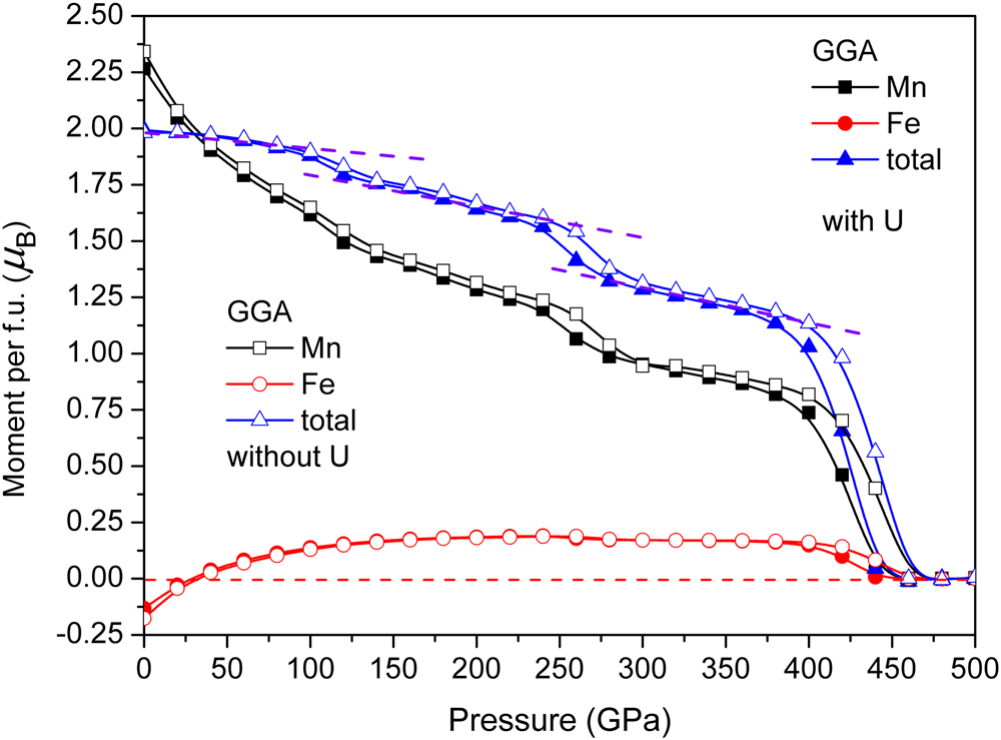
Magnetic moment per formula unit (f. u.) of Fe_2_MnAl at different pressures.

**Figure 10:**
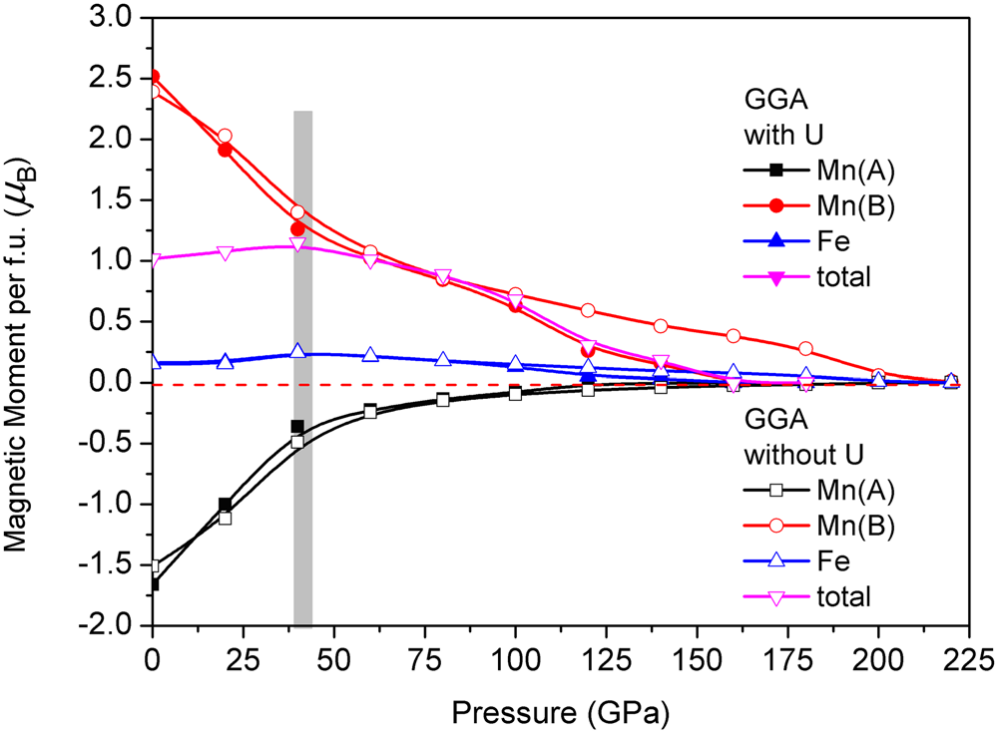
Magnetic moment per formula unit (f. u.) of Mn_2_FeAl at different pressures.

The total magnetic moments are 2.0^10,16,21^ and 1.0^14,15^ for Fe_2_MnAl and Mn_2_FeAl, respectively, consisting with the other calculations, as seen from Figs 9 and 10. The calculated results agree with the Slater-Pauling rule^22,23^. Calculated unit cell volume of Mn_2_FeAl is 193.1 Å^3^, which is 10.82 Å^3^ larger than that of Fe_2_MnAl, but the volume discrepancy is quickly reduced under compression and the volume of Mn_2_FeAl is always larger than that of Fe_2_MnAl at any pressure. However the volume shrinkage of Mn_2_FeAl is obviously faster than that of Fe_2_MnAl in the pressure range of 0–400 GPa.

The calculated zero-pressure moments are 2.25 and −0.125 *μ*_*B*_ for Mn and Fe in Fe_2_MnAl, respectively, in consistent with the other calculations (2.62, −0.31)^21^, (2.32, −0.15)^10^, (2.06, −0.06)^16^, (2.35, −0.16)^24^ in turn. The calculated moments are −1.66, 2.52, 0.16 *μ*_*B*_ for Mn (A), Mn (B), and Fe in Mn_2_FeAl, respectively, agreeing well with the other calculations (−2.54^14^, −1.85^15^), (3.42^2^, 2.81^15^), (0.06^14^, 0.05^15^) in turn. The values of Al are nearly zero and therefore could be ignored reasonably. Only one literature used *U* for Fe_2_MnAl^21^ but no one literature used it for Mn_2_FeAl.

**Figure 11:**
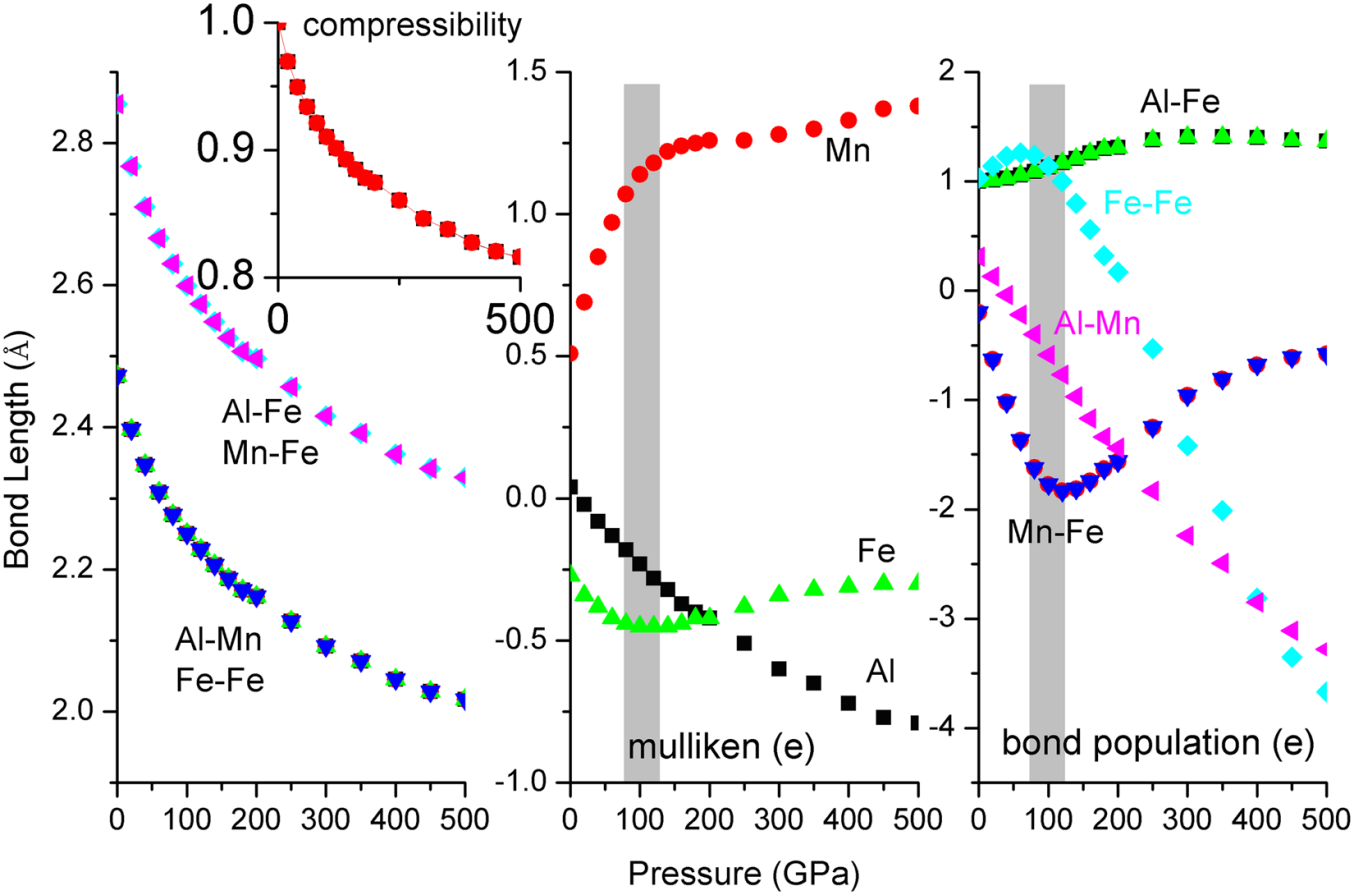
Bond length and its compressibility, mulliken charge, bond population of Fe_2_MnAl at different pressures.

**Figure 12:**
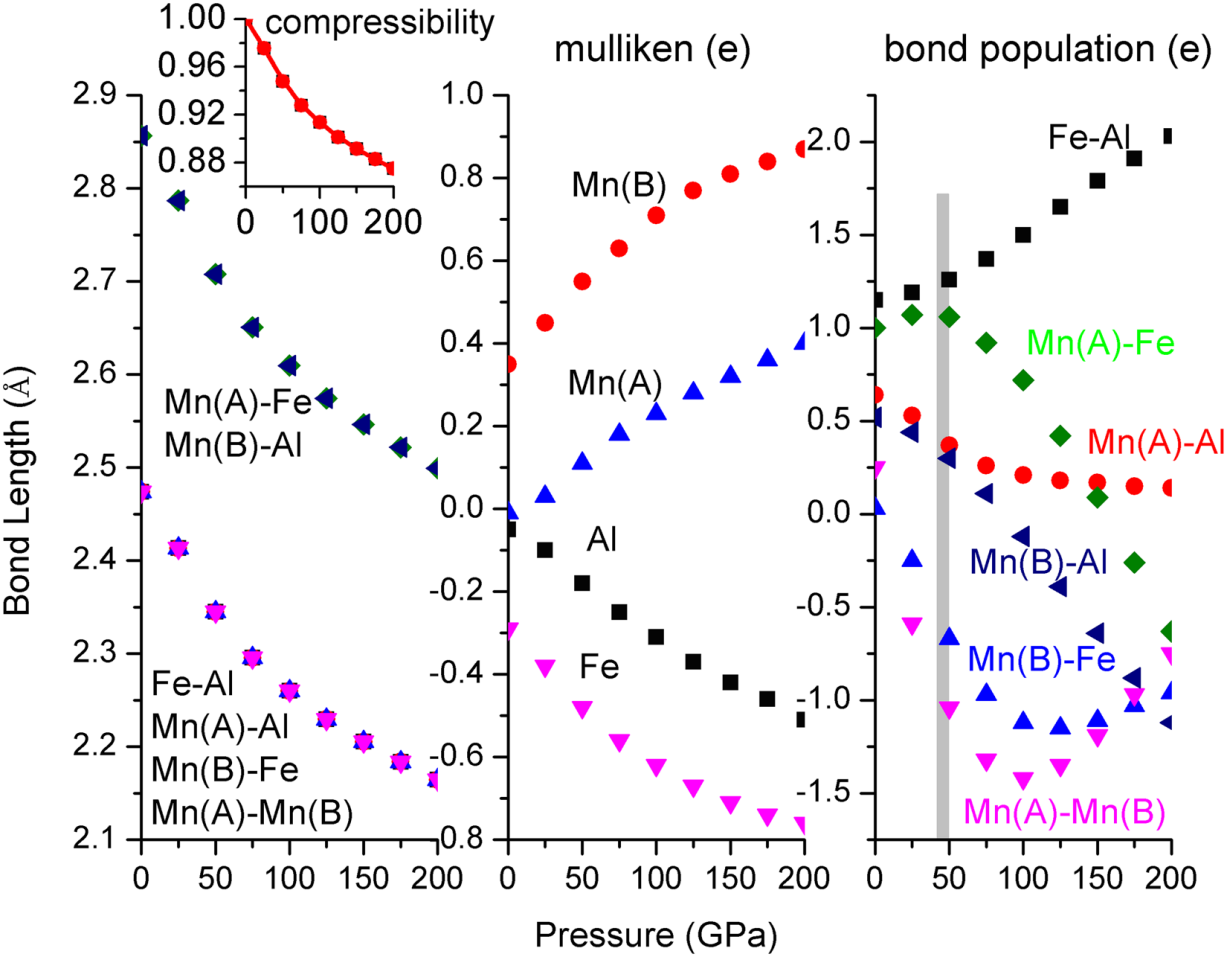
Bond length and its compressibility, mulliken charge, bond population of Mn_2_FeAl at different pressures.

The calculated moment collapse pressure is 450 GPa by GGA in Fe_2_MnAl. GGA results behave ladder-like decrease, existing three abrupt reductions at about 100, 250, 400 GPa, respectively. These three different slopes reveal the fast shift of spin-up and spin-down *d* state of Mn atom. The sudden collapse of Mn magnetism at 450 GPa indicates the instantaneous phase transition from ferromagnetic to nonmagnetic phases of Fe_2_MnAl, during which the cell volume and bond length didn’t occur any anomaly in Fe_2_MnAl, as are also the cases for Mn_2_FeAl, as shown in Figs 11 and 12. In addition, the compressibilities of the two different kinds of bonds are totally same, indicating the normal shrinkage of the geometry structure. Transition from high-spin to low-spin in MnO^25,26^ and MnGe^27^ are observed under pressure, in which moment collapse in MnGe^27^ is detected at about 5 GPa with the absence of abnormal moment enhancement behavior within 0–10 GPa. The mulliken charges and their bond populations of Fe and Mn behave critical phenomena at about 100 GPa, where the total moments of Fe_2_MnAl shows a sudden reduction. The charge transfer from Mn to Fe (and Al) slowdowns above about 100 GPa, above which the opposite charge transfer trend of Fe atom occurs, furthermore, the second collapse of total moment occurs at about 250 GPa, both of which are dominated by Mn atom. Accordingly, the two abrupt collapses originate mainly from the anomalous charge transfer under pressure. The rapid and giant collapse at about 400 GPa originates mainly from the energy band profile variation, which presents same variations with that of DOS. Within 0–100 GPa, the DOS of Fe decreases with pressure at Fermi level and keeps almost unchanged even at higher pressure in the spin-up channel, whereas the values of Mn increase with pressure and stabilize in a wide pressure range and increase again after the moment collapse. The DOS profiles of Fe and Mn clearly reflected their individually magnetic contributions to the total crystal structure. Both Fe *d* states, comprising of spin-up and spin-down components, distribute mainly at higher energy level, whereas Mn spin-up *d* states contribute mainly at lower energy level. Generally, both Fe *d* states shift towards lower energy level side under pressure, as is also the case in Mn. In fact, both Fe *d* states present highly delocalized features at 100 GPa below Fermi level, whereas this is not true in Mn, indicating that Mn also plays a key role to the total magnetism at high pressure.

The moment direction of Fe is changed at about 25 GPa in Fe_2_MnAl, and its magnitude keeps almost unchanged between 50 GPa and 400 GPa, with a value of about 0.15 *μ*_*B*_. The magnetism collapse process is highly correlated with the mulliken charge and bond population distributions, through which many evident evidences can be clearly seen, such as a strong indicator of charge transfer tendency is seen at the first collapse pressure 100 GPa, as are also the cases of 250 and 400 GPa, respectively.

The total moment curve of Mn_2_FeAl shows a peak at about 40 GPa, which is similar with the variation tendency of Mn(A)-Fe bond population. In addition, Mn(A/B)-Al also changed their curve slopes at 40 GPa. Moreover, opposite bond population trends in Mn(A)-Mn(B) and Mn(B)-Fe are formed at 100 GPa, suggesting the strong correlation of the bond population and the moment.

Generally, the magnetism characters of Mn and Fe consist with their respective DOS features. Moreover, the total DOS profile agrees with the energy band, namely, the energy gap of spin-down channel is closed at 50 GPa in Mn_2_FeAl and 100 GPa in Fe_2_MnAl. Fe *d* states occupy higher energy level sites and Mn *d* states distribute mainly at lower sites in Fe_2_MnAl. The DOS curves of Mn in both compounds consist with its magnetic direction. Under pressure, Mn(A) and Mn(B) display contrary magnitude variations at Fermi level at spin-up channel in Mn_2_FeAl, whereas Mn(B) varies larger than that of Mn(A) at spin-down channel.

## Conclusions

The present investigation demonstrates that the Fe_2_MnAl and Mn_2_FeAl are highly stable under pressure, suggesting the impossibility of structural phase under pressure at least up to 400 GPa in Fe_2_MnAl and Mn_2_FeAl. The unusual moment evolution under pressure reveals the unusual charge transfer and bond population rearrangement. There are three moment collapses in Fe_2_MnAl and one abnormal moment increase in Mn_2_FeAl under pressure. These noncontinuous and continuous variations in Fe_2_MnAl and Mn_2_FeAl mean the spin state transition under pressure. The atomic density of states of Fe_2_MnAl and Mn_2_FeAl shifts towards lower energy side under pressure. The magnitudes of total density of states of Fe_2_MnAl and Mn_2_FeAl keep almost unchanged at Fermi level in a wide pressure range at spin-up channel.
